# Redox characterisation of Erv1, a key component for protein import and folding in yeast mitochondria

**DOI:** 10.1111/febs.15136

**Published:** 2019-11-29

**Authors:** Efrain Ceh‐Pavia, Xiaofan Tang, Yawen Liu, Derren J. Heyes, Bing Zhao, Ping Xiao, Hui Lu

**Affiliations:** ^1^ Faculty of Biology, Medicine and Health School of Biological Sciences University of Manchester UK; ^2^ School of Materials University of Manchester UK; ^3^ State Key Laboratory of Supramolecular Structure and Materials Jilin University Changchun China; ^4^ Manchester Institute of Biotechnology University of Manchester UK

**Keywords:** FAD‐dependent enzyme, MIA pathway, reduction potential, sulfhydryl oxidase

## Abstract

The mitochondrial import and assembly (MIA) pathway plays a vitally important role in import and oxidative folding of mitochondrial proteins. Erv1, a member of the FAD‐dependent Erv1/ALR disulphide bond generating enzyme family, is a key player of the MIA pathway. Although considerable progress has been made, the molecular mechanism of electron transfer within Erv1 is still not fully understood. The reduction potentials of the three redox centres were previously determined to be −320 mV for the shuttle disulphide, −150 mV for the active‐site disulphide and −215 mV for FAD cofactor. However, it is unknown why FAD of Erv1 has such a low potential compared with other sulfhydryl oxidases, and why the shuttle disulphide has a potential as low as many of the stable structural disulphides of the substrates of MIA pathway. In this study, the three reduction potentials of Erv1 were reassessed using the wild‐type and inactive mutants of Erv1 under anaerobic conditions. Our results show that the standard potentials for the shuttle and active‐site disulphides are approximately −250 mV and −215 ~ −260 mV, respectively, and the potential for FAD cofactor is −148 mV. Our results support a model that both disulphide bonds are redox‐active, and electron flow in Erv1 is thermodynamically favourable. Furthermore, the redox behaviour of Erv1 was confirmed, for the first time using Mia40, the physiological electron donor of Erv1. Together with previous studies on proteins of MIA pathway, we conclude that electron flow in the MIA pathway is a thermodynamically favourable, smoothly downhill process for all steps.

**Database:**

Erv1: EC 1.8.3.2.

## Graphical abstract

The mitochondrial import and assembly (MIA) pathway is required for the oxidative folding and import of mitochondrial intermembrane space proteins. In this study, we examined the redox properties of Erv1, an essential component of the MIA pathway, to shed light on the molecular mechanism of electron transfer. Electron flow is a thermodynamically favourable downhill process over all steps in the MIA pathway. Interestingly, the redox potential of Erv1 active‐site disulphide depends on the redox state of the shuttle disulphide bond.

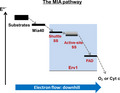

**Abbreviations**
ALR: augmenter of liver regeneration
DTT: dithiothreitol
Erv: essential for respiration and viability
FAD: flavin adenine dinucleotide
IMS: intermembrane space
MIA: mitochondrial import and assembly
QSOX: quiescin sulfhydryl oxidase
TCEP: tris(2‐carboxyethyl)phosphine

## Introduction

All the mitochondrial intermembrane space (IMS) proteins are synthesised in the cytosol and most of them contain conserved Cys residues. Consequently, disulphide bond formation plays a key role during the biogenesis of these IMS proteins 1, 2. They are synthesised in a Cys‐unfolded and Cys‐reduced form that is imported into mitochondrial IMS via a redox‐sensitive pathway, called the mitochondrial import and assembly (MIA) pathway 3, 4, 5, 6. Erv1 (essential for respiration and viability 1) in yeast or called ALR (augmenter of liver regeneration in mammals 7, 8, 9) is an essential component of the MIA pathway, and together with Mia40, catalyses oxidative folding of the newly imported IMS proteins 4, 5. Upon mitochondrial protein import, Mia40 acts as an oxidoreductase interacting with the substrate proteins directly and transfers a disulphide bond to the substrates via the formation of intermolecular disulphide linked complexes 10, 11, 12, 13. The reduced Mia40 is then re‐oxidised by Erv1 (EC 1.8.3.2) for regeneration, and reduced Erv1 can be re‐oxidised by molecular oxygen or oxidised cytochrome *c* 14.

**Figure 1:**
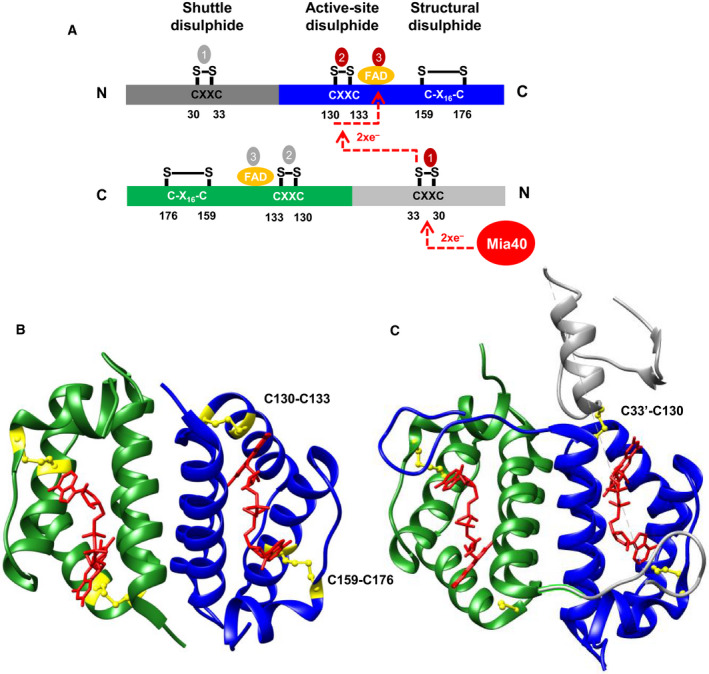
Structure and redox centres of yeast Erv1. (A) Schematic structure of Erv1 with electron flow from Mia40 to Erv1 and between the three redox centres (red/grey cycles 1–3) of Erv1 dimer shown. (B) X‐ray structure of the C‐terminal domain of Erv1 (the PDB code is 4E0H) 19 with the active‐site (proximal) disulphide and the structural disulphide shown in yellow, and FAD shown in red stick. (C) X‐ray structure of Erv1C30,133S mutant (the PDB code is 4E0I) 19 with interdomain intermediate C33’‐C130 disulphide and the structural disulphide shown in yellow, and FAD shown in red stick. The protein structures were produced using chimera 1.14 program (San Francisco, CA, USA). PDB, Protein Data Bank.

Erv1 belongs to the Erv/ALR sulfhydryl oxidase family, having been identified in all mitochondria containing eukaryotes 8, 15, 16. All these enzymes form a dimer and possess a highly conserved catalytic core domain of ~ 100 amino acids folded in a helix bundle and stabilised by noncovalently binding of flavin adenine dinucleotide (FAD) (Fig. 1) 17, 18, 19. The catalytic (or FAD‐binding) domain of yeast (*Saccharomyces cerevisiae)* Erv1 is at the C terminus. The FAD‐binding domain also contains a redox active‐site CXXC disulphide bond (Cys130‐Cys133), which is also known as the proximal disulphide as it is located proximal to the isoalloxazine ring of FAD, and a CX_16_C structural disulphide (Cys159‐Cys176) 12, 20, 21. Furthermore, Erv1 has a functionally important shuttle disulphide bond (Cys30‐Cys33) in the nonconserved and unfolded N‐terminal domain 12, 22. Studies have shown that the shuttle disulphide bond of Erv1 can be reduced by reduced Mia40 or disulphide reducing agents, for example dithiothreitol (DTT) and tris(2‐carboxyethyl)phosphine (TCEP) 12, 22. The electrons are then transferred to the active‐site (proximal) disulphide via formation of an intermediate disulphide (Fig. 1C, C33’‐C130), the cofactor FAD, and then in turn the reduced Erv1 can be re‐oxidised by transferring electrons to molecular oxygen (O_2_) or cytochrome *c* for regeneration 14, 23, 24. Thus, there are three redox‐active centres in Erv1, the shuttle disulphide (C30‐C33), active‐site (proximal) disulphide (C130‐C133) and the FAD cofactor. In addition, the Erv/ALR FAD‐binding domain also exists in the endoplasmic reticulum of yeast (Erv2) 25, in poxviruses (E10R) 26 and in sulfhydryl oxidases of the extracellular environment (QSOX: quiescin sulfhydryl oxidase) 27.

The reduction potential (E^0^′) of a redox compound is a key parameter for understanding electron transfer pathways in redox reactions. The higher oxidising power, the higher the reduction potential (less negative) is. A previous study by Dabir *et al.* 24 reported the three reduction potentials of Erv1 to be −320 mV for the shuttle disulphide, −150 mV for the active‐site disulphide and −215 mV for the FAD cofactor, respectively. However, the results raised some important questions. For example, why does the FAD of Erv1 have such a low (−215 mV) potential compared with other sulfhydryl oxidases 9, 18, 28? These potential values would suggest that the electron flow from the active‐site (proximal) disulphide to FAD was a thermodynamically unfavourable process in Erv1. Although very few studies have been reported, a similar phenomenon has not been observed for other well‐characterised Erv/ALR enzymes, such as Erv2 and ALR 9, 18. However, the potential of Erv1 shuttle disulphide, located in the unfolded N‐terminal domain, was shown to be −320 mV, suggesting it is a surprisingly stable disulphide bond and inconsistent with its function of acting as a redox active shuttle disulphide. It should be noted that the wild‐type enzyme was used in the previous redox titration experiments 24. This is likely to affect the measurements as the enzyme was active and would have altered the redox potential of the redox buffers, for example turned DTT to oxidised DTT (oxDTT) during sample incubation. Thus, it is important to re‐evaluate the redox properties of Erv1 as it is a key and essential component for mitochondrial protein biogenesis.

To understand whether yeast Erv1 does have unique redox properties and to understand the thermodynamics of the electron flow in Erv1, in this study, we re‐examined redox properties of yeast Erv1. The reduction potentials of the three redox centres in Erv1 were determined for the wild‐type and an inactive double Cys mutant of Erv1. In addition, Erv1 redox titrations were carried out under anaerobic conditions using both chemical reductants and, for the first time, Mia40, the physiological electron donor of Erv1. Our results showed that the FAD cofactor has the most positive (less negative) reduction potential (−148 mV) of the three redox centres of Erv1, and the two disulphide bonds have a very similar value of reduction potential (about −215 ~ −260 mV). Together with previous studies, we conclude that electron flow in the MIA pathway is a smoothly downhill process over all the steps.

## Results and Discussion

### Electron titration of Erv1 under anaerobic conditions

**Figure 2:**
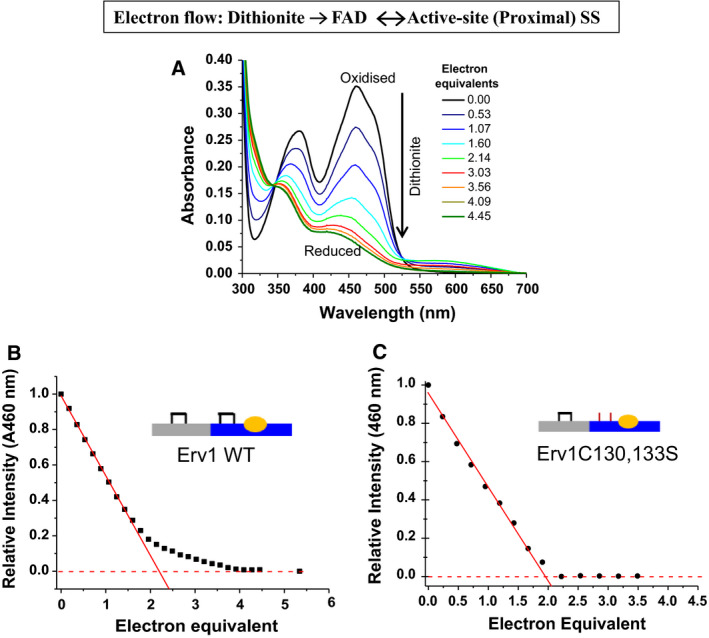
Electron titration of FAD in Erv1. (A) UV‐visible absorbance spectra of Erv1 recorded before (black line) and after addition of sodium dithionite at various electron equivalents (electrons/FAD) under anaerobic conditions. Absorbance spectra of FAD at fully oxidised (black) and fully reduced (olive green) were shown in thick lines. (B) Normalised absorbance at 460 nm vs electron equivalents for the data shown in A. The red line represents a linear fit to the early data points with a slope of −0.49. About four electrons were required to fully reduce FAD. The value represents the average of two independent experiments. (C) As in (B) but normalised sodium dithionite titration curve for Erv1C130,133S mutant. The red line represents a linear fit to the early data points with a slope of −0.49. About two electrons were required to reduce FAD.

In order to know how many electrons are required to fully reduce the FAD cofactor of Erv1, a dithionite titration under anaerobic conditions was performed and followed by UV‐Vis spectra measurement (Fig. 2). Methyl viologen was added to mediate the transfer of reducing equivalents between dithionite and FAD of Erv1. The absorbance intensity of the FAD decreases at 460 nm upon reduction and was plotted against the number of electron equivalents (Fig. 2B). The decrease showed a linear dependence on the number of electrons with about 85% FAD reduction followed a linear line with a slope of −0.49. Thus, approximately 2 electrons were transferred per FAD initially. Subsequently, the FAD reduction became less efficient and (at least) another two electrons were required for the remaining ~ 15% FAD. Hence, four‐electron equivalents of dithionite are required to fully reduce the two‐electron acceptor FAD. On the other hand, only two electrons were required to reduce the Erv1C130,133S double Cys mutant as shown in Fig. 2C. These results suggest that not only the FAD of Erv1 was reduced, but the proximal redox‐active disulphide bond was reduced as well during the later stage of the titration of the WT Erv1. Our results also showed that the reduction potential of Erv1 proximal disulphide could be determined using dithionite titration. It should be noted that the appearance of the absorption peak at about 550–650 nm indicates the presence of the blue neutral semiquinone of FAD, similar to that of ALR 9.

### Reduction potential of the cofactor FAD

**Figure 3:**
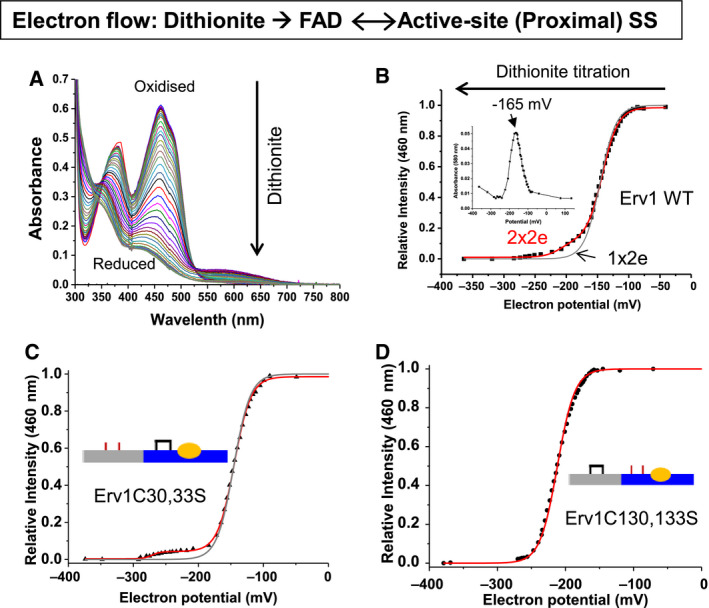
FAD redox potential titration of Erv1. (A) Representative UV‐visible absorbance spectra of Erv1 recorded at various electron potentials during dithionite titration under an anaerobic condition. FAD goes from fully oxidised (top, black line) to fully reduced (bottom, olive green line). (B) Normalised absorbance at 460 nm vs electron potential for the WT Erv1 data shown in A. The data were fitted using Nernst equation for one 2‐electron (1x2e, black line) and two 2‐electron (2x2e, red line) reactions, respectively. A standard reduction potential of −150 ± 5 mV for 1x2e was obtained, whilst two standard reduction potentials of −148 ± 5 and −215 ± 5 mV for 2x2e were determined. The values represent average of two independent experiments. The insert is a plot of intensity at 580 nm vs electron equivalents for the data shown in A. (C) As described in B but for Erv1C30,33S mutant. The data were best fitted with a Nernst equation for 2x2e reaction with two reduction potentials of −145 and −271 mV obtained (red line). (D) As in described in B but for Erv1C130,133S mutant. The data were best fitted to a Nernst equation for a 1x2e reaction with a reduction potential of −212 mV obtained (red line).

After it was established that both the FAD and the proximal disulphide (C130‐C133) of Erv1 could be reduced by dithionite titration, we repeated the titration experiment with more data points and in the presence of a range of redox mediators (see Materials and methods) to simultaneously measure both the electron potential and UV‐visible absorbance spectra (Fig. 3A). The relative absorbance intensity at 460 nm was plotted against electron potential, and the data were analysed using the Nernst equation (Fig. 3B). The analysis gave an apparent midpoint potential E_m_ of −150 ± 5 mV for a 2‐electron reduction of Erv1 (black line). Clearly, not all the data points can be fitted to the equation single 2‐electron reaction, it may partially due to the accumulation of semiquinone intermediate (Fig. 3B, insert). In particular, when redox potential was more reducing (negative) than −160 mV, the data points were clearly away from the fitted line. This result was consistent with the above conclusion that the proximal disulphide was reduced in parallel with the FAD at the later stage of FAD reduction (Fig. 2B). Furthermore, we analysed the data with Nernst equation for a two 2‐electron (2x2e) reaction, which fitted the data very well (red line) with two standard reduction potentials of −148 ± 5 mV (FAD) and −215 mV ± 5 mV (disulphide bond) were determined. Moreover, the same experiment was performed with Erv1C30,33S (Fig. 3C) and Erv1C130,133S (Fig. 3D) double Cys mutants. Whilst two reduction potentials of −145 mV (for FAD) and −271 mV (disulphide bond) were determined for Erv1C30,33S, one reduction potential of −212 mV (for FAD) was determined for Erv1C130,133S. The results confirmed that (a) it was the shuttle disulphide bond that was reduced during the later stage of FAD reduction, and (b) mutation of the proximal disulphide (C130‐C133), but not the shuttle disulphide, affected the reduction potential of FAD cofactor. Thus, we signed the potentials of −148 ± 5 mV for FAD of Erv1. This reduction potential is similar to that reported for human sfALR (−178 mV), QSOX (−153 mV), and it is a bit more positive than that reported for Erv2 (−200 mV), an ER localised sulfhydryl oxidase in yeast 9, 18, 28.

### Reduction potential of the proximal disulphide

**Figure 4:**
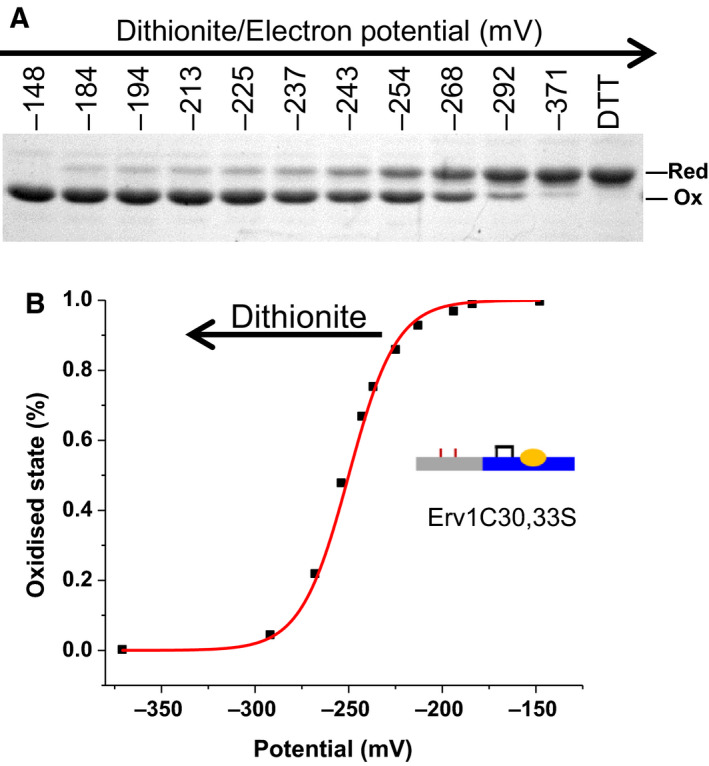
Redox state analysis of the proximal disulphide bond of Erv1 with Erv1C30,33S mutant. (A) SDS/PAGE of an AMS assay of Erv1C30.33S taken at various potentials along a FAD dithionite titration under an anaerobic condition. Positive control: the protein was treated with 1 mm DTT and then 10 mm AMS. (B) Quantification of data shown in A and analysed use Nernst equation with a reduction potential of −260 ± 5 mV was obtained from two independent experiments.

A proximal disulphide in direct redox communication with the FAD isoalloxazine is a key feature shared by all the disulphide oxidoreductases. The reduction potential of this disulphide is hard to measure due to its active communication with the FAD cofactor. Based on our results above (Fig. 3), the reduction potential of C130‐C133 was estimated to be between −200 and −300 mV. To verify this, we repeated the titration with Erv1C30,33S, and the redox state of the proximal disulphide bond (C130‐C133) was analysed using AMS thiol‐modification assay. As shown in Fig. 4, a small fraction of Erv1 was reduced start from reduction potential of about −180 mV and more was reduced with the addition of dithionite hence at more negative potential. An apparent reduction potential of −260 ± 5 mV was determined for the proximal disulphide bond (C130‐C133), similar to that determined from Fig. 3C. Thus, the results of our two independent methods showed that the reduction potential of the proximal disulphide is at about −215 mV (in the WT, C30‐C33 disulphide bonded) to −260 mV (in C30.33S mutant) with an average of −238 mV. It is ~ 90 mV more reducing (negative) than the FAD of Erv1 (−148 mV). There is a clear difference between the results obtained by using the WT (−215 mV) and Erv1C30,33S mutant (−260 mV), which may be caused by changes in protein conformation and/or interactions between the N‐terminal and C‐terminal domains due to C30,33S mutation. Without the shuttle disulphide bond, the flexible N‐terminal domain could exist in a different conformation and be primed for interaction with the proximal disulphide, which may result in the potential of the proximal disulphide shifted shifting from −215 to −260 mV. Thus, our results showed that the proximal disulphide bond (C130‐C133) has a reduction potential about −215 (−215 mV ~ −260 mV), and interestingly depends on the redox state or conformation of the shuttle disulphide bond. This is consistent with our result that four (not six) electrons are required to reduce Erv1 FAD fully (Fig. 2B).

It is experimentally challenging to determining the potential of an active‐site (proximal) disulphide of Erv1/ALR or Erv domain‐containing enzyme due to its activity. It was determined to be −235 mV for the human sfALR 29 and −273 mV for QSOX 28 by using the more reducing FAD homologue 5‐deaza‐FAD substituted enzymes. These values are similar to our result of Erv1 proximal disulphide (−215 ~ −260 mV) determined in this study.

In summary, in contrast to the previous report by Dabir *et al*. 24 that the proximal disulphide (C130‐C133) of Erv1 was less reducing than FAD, our results show that C130‐C133 is more reducing and thus electron flow from the proximal disulphide to the FAD is a thermodynamically favourable downhill reaction. Our conclusion is consistent with that of human ALR and QSOX that were both reported to perform a thermodynamically favourable downhill reaction during electron flow from the proximal disulphide to the FAD cofactor.

### Reduction potential of the shuttle disulphide

**Figure 5:**
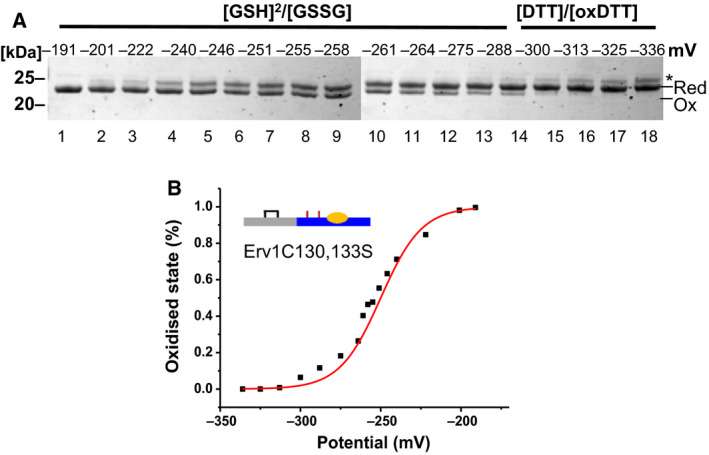
Redox state analysis of the shuttle disulphide bond of Erv1 with Erv1C130,133S mutant. (A) SDS/PAGE of an AMS assay of Erv1C130,133S taken after incubation in a redox buffer at various redox potentials (lanes 2–17). Lane 1: Oxidised control of untreated protein; lane 18: Reduced control of the protein pretreated with 1 mm DTT and then 10 mm AMS. The * indicates a band with the structural disulphide bond reduced as well. (B) Quantification of data shown in A and analysed using Nernst equation with a reduction potential of −250 ± 5 mV was obtained based on two independent experiments. Each corresponding potential was calculated based on using [GSH]^2^/[GSSG] or [DTT]/[oxDTT] in the Nernst equation.

A solvent‐accessible redox‐active disulphide bond often has a reduction potential around −200 mV. Such as the E^0^′ of glutathione (GSH/GSSG) is −240 mV at pH 7.0, about −120 mV for the most oxidising DsbA CXXC disulphide, and about −270 mV for the most reducing Trx CXXC disulphide 30, 31, 32. On the other hand, many substrate proteins of the MIA pathway have reduction potentials around −310 to −340 mV for their stable structural disulphides 33, 34, 35, 36, 37, and it is about −290 mV for yeast Mia40 CPC 37. The previously measured reduction potential of the Erv1 shuttle disulphide was −320 mV 24, which seems to be too stable and inconsistent with its function as shuttle disulphide bond 12. In this study, we re‐measured the reduction potential of the Erv1 shuttle disulphide with the inactive Erv1C130,133S double Cys mutant coupled with the AMS assay (Fig. 5). Redox buffer at various potentials was prepared using GSH/GSSG or DTT/oxDTT as described previously 24. After incubation for 3 h, the samples were TCA precipitated before AMS assay and analysed using nonreducing SDS/PAGE (Fig. 5). A reduction potential of −250 mV was obtained for the shuttle disulphide of Erv1, which is slightly more negative but similar to that of the active‐site (proximal) disulphide (−215 ~ −260 mV). Our result of the shuttle disulphide is clearly different from −320 mV published by Dabir *et al*. 24. We reasoned this difference may be due to different proteins (WT vs. C130,133S mutant) or data analysis. To address this, we reanalysed our data using [GSH]/[GSSG] rather than [GSH]^2^/[GSSG] in the Nernst equation and a reduction potential of −318 mV was obtained, which may explain the difference. We also tried to repeat the measurement with WT Erv1, but did not get a clear result, possibly (partially) due to recycling of the active enzyme. On the other hand, the difference may also reflect a redox change due to conformation change caused by the C130,133S mutation. This is less likely as the proximal Cys residues are located in a folded domain and the shuttle Cys are in an unfolded domain, and a reduction potential of −320 mV is too stable for disulphide bond in unfolded protein domain.

### Verification of Erv1 redox parameters using reduced Mia40 as electron donor

**Figure 6:**
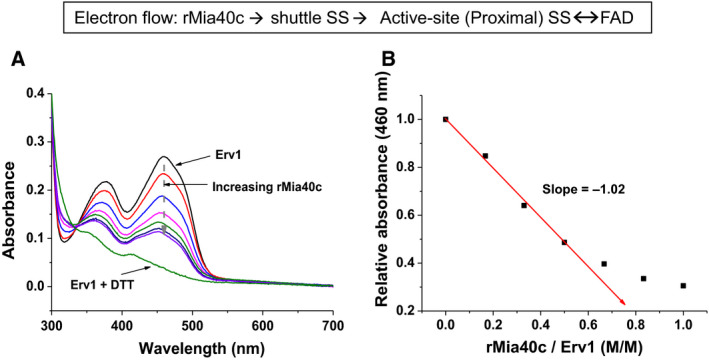
Reduction of Erv1 by rMia40c titration under anaerobic condition. (A) UV‐Via Spectra of Erv1 in the absence (top, black line) and presence of various amount of rMia40c, together with the FAD fully reduced spectra (Erv1 + DTT, olive green). (B) Plot of normalised absorbance at 460 nm vs the molar ratio of rMia40/Erv1 for the data shown in A. The normalisation was based on the intensity of fully oxidised Erv1 (in the absence of rMia40c) as 1 and the DTT reduced Erv1 as 0. The red line represents a linear fit with the early data points of the titration, with a slope of 1.0 obtained.

Dithionite is a convenient and commonly used chemical electron donor for FAD reduction studies. In practice, Mia40 is the physiological electron donor or upstream substrate of Erv1/ALR enzymes in mitochondria, and it reduces Erv1 via the shuttle and active‐site (proximal) disulphide bonds to FAD (Fig. 6). Since our result is different from the previous result by Dabir *et al*. 24 in terms of the relative reduction potentials between the proximal disulphide and FAD cofactor, we carried out Erv1 reduction titration using reduced Mia40. The functional C‐terminal domain of Mia40 was reduced (rMia40c) by incubation of the purified protein with 0.5 mm TECP as described previously 21, and followed by buffer exchange inside an anaerobic glove box to remove TCEP and molecular oxygen. The UV‐visible spectra of Erv1 were measured during rMia40c titration and are shown in Fig. 6. The absorbance of the FAD decreased with the increase of rMia40c linearly with a slope of about 1.0 until about 60% FAD was reduced, showing that the two electrons from rMia40c flowed to the FAD at a molar ratio of 1 : 1 initially. The remaining FAD reduction was slowed down consistent with above observation of reduction of the FAD and disulphide bonds in parallel. These results confirmed that the FAD cofactor has the most positive (less negative) reduction potential amongst the three redox centres of Erv1. Furthermore, no peak around 580 nm was observed during rMia40c titration, and thus, no semiquinone accumulation was detected when the physiological substrate Mia40 was used to reduce Erv1. This result suggests that the blue neutral semiquinone accumulation observed during dithionite titration of Erv1 and many other FAD‐dependent enzymes may be an artefact of chemical reduction, which may need to be re‐evaluated in some cases.

## Conclusion

**Figure 7:**
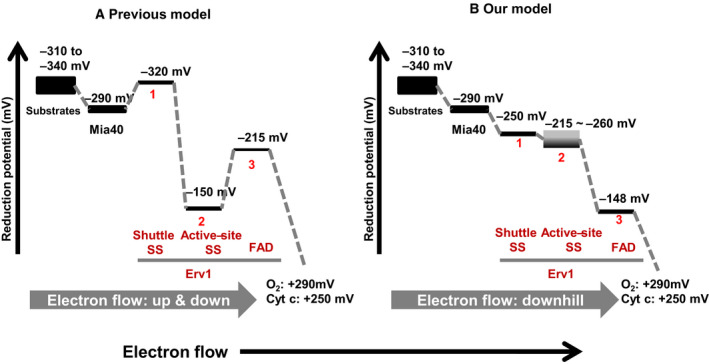
Schematic energetic landscape of the MIA pathway. The reduction potentials for known substrates, Mia40 and three redox centres of Erv1 are shown. In the previous model (A), electron flows up and down between Mia40 and Erv1 and within Erv1. In our model (B), electron flows smoothly downhill over all the steps of the MIA pathway.

The overall aim of this study was to investigate the thermodynamic mechanism regulating the electron flow in the mitochondrial sulfhydryl oxidase Erv1. The reduction potentials of three redox centres in Erv1 were determined to be: −250 ± 5 mV for the shuttle disulphide bond (C30‐C33), −215 to −260 mV for the proximal disulphide bond (C130‐C133) and −148 ± 5 mV for the FAD cofactor (Fig. 7). Interestingly, the redox potential of the proximal disulphide seems to depend on the redox state of the shuttle disulphide bond. Furthermore, the physiological substrate Mia40 was used to confirm that the FAD has the most positive (less negative) reduction potential in Erv1, and no semiquinone accumulation was observed during the Mi40‐Erv1 reduction. Together with previous studies, we conclude that electron flow in the MIA pathway is a thermodynamically favourable smoothly downhill process over all the steps, from the upstream imported substrate proteins to Mia40‐Erv1 and to the downstream electron acceptors (Fig. 7).

## Materials and methods

### Materials

All chemicals used in this study were analytical grade and were from Sigma‐Aldrich Inc. (St. Louis, MO, USA) or Thermo Fisher Scientific (Waltham, MA, USA) unless specified. All solutions prepared using MilliQ water. Unless specifically stated, all experiments in this study were carried out in a buffer called BAE (50 mm Tris/HCl, 150 mm NaCl, 1 mm EDTA, pH 7.4) at 25 °C.

### Protein purification

For Erv1 (the WT and mutants), the pET‐24a(+) plasmid containing yeast ERV1 gene was expressed in *Escherichia coli* Rosetta‐gami™ 2 cells (Novagen, Merck KGaA, Darmstadt, Germany) and purified using Ni‐NTA (Ni^2+^‐nitrilotriacetate) His‐bind beads (Novagen) as described previously 12, 21, 38. Further purification was done by size‐exclusion chromatography (SEC) using buffer AE (BAE: 50 mm Tris/HCl, 150 mm NaCl and 1 mm EDTA, pH 7.4) on a Superdex 200 or Superdex 200plus 100/300 GL column (GE Healthcare Bio‐sciences, Uppsala, Sweden). Similarly, Mia40c (C‐domain of Mia40: residues 284–403) was expressed in *Escherichia coli* Rosetta‐gami™ 2 cells (Novagen) and purified using Ni‐NTA beads 21, 39. Superdex 75 100/300 GL column was used to further purify and isolate the monomer proteins for this study.

### Preparation of reduced Mia40c (rMia40c)

Purified Mia40c was incubated with 0.5 mm Tris(2‐carboxyethyl)phosphine (TCEP) for at least 10 min at 25 °C as described previously 21. Excess TCEP was removed using a PD‐10 column pre‐equilibrated with anaerobic buffer BAE (BAE: 50 mm Tris/HCl, 150 mm NaCl, 1 mm EDTA, pH 7.4) inside an anaerobic glove box (Belle Technology, Weymouth, UK), with oxygen levels maintained below 2 p.p.m. Buffers were made anaerobic by extensive bubbling with oxygen‐free nitrogen, prior to incubation inside the anaerobic glove box. The concentration of rMia40c was determined using a ε280 of 11.7074 mm ^−1^·cm^−1^ as predicted using the protparam software.

### Erv1 reduction under anaerobic conditions

For all anaerobic experiments, the assays were done within an anaerobic glove box (Belle Technology), with oxygen levels maintained below 2 p.p.m. Buffers were made anaerobic by extensive bubbling with oxygen‐free nitrogen, followed by incubation inside the anaerobic glove box. Proteins were buffer exchanged using PD‐10 column to anaerobic BAE in an anaerobic glove box, before each experiment.

### FAD electron titration with dithionite

Electron titrations of the FAD cofactor in Erv1 were conducted in BAE using protein concentrations (based on bound FAD) ranging from 30 to 60 μm. A freshly made sodium dithionite solution was titrated using an anaerobic FAD solution of known concentration (ε450 = 11.3 mm ^−1^·cm^−1^). This titration was done before and after the Erv1 assays to obtain an average normality. Small known volumes (0.5–2 μL) of dithionite solution were then used to reduce the protein. The UV‐visible spectrum was recorded from 250 to 700 nm after each addition (equilibration time of 10–15 min) using a Cary 50 Bio UV‐visible spectrophotometer. The point at which the reduction of FAD was completes (no further decrease in absorbance at either 460 marked the number of electrons required for complete FAD reduction. The relative absorbance change at 460 was normalised with the value of the fully oxidised FAD set as 1 and the value of the fully reduced FAD as 0. The results are the average of two independent experiments.

### FAD reduction potential titration with dithionite

Electron potential measurements were conducted in BAE with protein concentrations (based on bound FAD) of 30–80 μm. Mediators for improved conductivity between the protein and electrode were added. Typically, these were (final concentrations) 2 μm phenazine methyl sulphate [E_1/2_ 80 mV vs normal hydrogen electrode (NHE)], 7 μm 2‐hydroxy‐1,4‐naphthoquinone (E_1/2_ −145 mV vs NHE), 1 μm benzyl viologen (E_1/2_ −311 mV vs NHE) and 0.3 μm methyl viologen (E_1/2_ −430 mV vs NHE). Sodium dithionite solutions (in BAE) were made up fresh inside the glove box immediately before the experiment. Small volumes (0.5–5 μL) of the solution were gradually added to reduce the protein (5 mL). Adequate time (10–20 min) was allowed for electronic equilibration following each addition of reductant, and prior to the spectrum being recorded at a stabilised reduction potential. Spectra were recorded from 300 to 800 nm using a fibre optic probe (Varian) immersed in the protein solution and connected to a Cary 50 Bio UV‐visible spectrophotometer. The electron potential was monitored using a Seven Easy pH meter (METTLER TOLEDO, Leicester, UK) with a Pt/Calomel electrode and a correction factor of +240 mV to adjust the electron potential. The protein was mixed slowly throughout the assay using an 8.5 mm magnetic flea on a magnetic stirrer.

The relative absorbance change at 460 was normalised with the value of the fully oxidised FAD set as 1 and the value of the fully reduced FAD as 0. To calculate the standard redox potentials, the collected data were fitted to the Nernst equation with the number of electrons restricted to *z* = 2. For two 2‐electron reaction analysis, the following equation was used:

$$Y=\frac{A1\times 10^{X-E1\times (2/59)}+A2+A3\times 10^{E2-X\times (2/59)}}{1+10^{X-E1\times (2/59)}+10^{E2-X\times (2/59)}}.$$

where A1–A3 are amplitudes of relative absorbance change, E1 and E2 are the standard reduction potentials. The results represent the average of three independent experiments. The fittings were done using the Origin 8.5 software.

### AMS thiol modification assay

Thiol‐disulphide redox state was analysed using the agent 4‐acetamido‐4′‐maleimidylstilbene‐2,2′‐disulphonic acid (AMS). The thiol‐modifier AMS (Molecular Probes, Thermo Fisher Scientific) covalently reacts with any free thiols resulting in an increase in the molecular weight of 0.5 kDa per thiol. For the proximal disulphide potential determination, Erv1C30,33S mutant was used. The protein samples were taken along the dithionite redox titration experiment and mixed with an equal volume of anaerobic 2X nonreducing SDS/PAGE sample buffer containing 2 mm AMS. The positive control for complete reduction of the Erv1 disulphide was made by incubating the protein with 1 mm DTT under anaerobic condition for 10 min followed by incubation with 2 mm AMS and nonreducing sample buffer, in dart for about 10 min. For the shuttle disulphide potential determination, Erv1C130,133S was used. A set of redox buffers from −180 to −360 mV was prepared using various rations of reduced/oxidised glutathione (GSH/GSSG) or DTT/oxDTT as described previously 24. The potentials of the redox buffers were calculated using Nernst equation and [GSH]^2^/[GSSG] or [DTT]/[oxDTT], respectively. The E_h_^0’^ of −264 mV for GSH/GSSG and −354 mV for DTT/oxDTT at pH 7.4 was used. The proteins were incubated for about 3 h in the redox buffers saturated with nitrogen gas. After incubation for about 3 h, the reaction was quenched and the proteins were precipitated by the addition of TCA (10%) on ice for 30 min. The protein pellets were washed with acetone. Then, the precipitated proteins were solubilised and incubated in a buffer containing 2 mm AMS and nonreducing SDS/PAGE sample buffer, in dart for about 10 min. All samples were analysed using 16% tris‐tricine SDS/PAGE, coomassie staining and band intensity qualification using imagej program.

## Conflicts of interest

The authors declare no conflict of interest.

## Author contributions

All authors have contributed to experimental design and data analysis. ECP performed the experiments for Figs 2, 3, 4; XT help with data analyse for Fig. 3 and did the experiments for Fig. 6; YL did the experiments for Fig. 5. DJH helped ECP and XT technically with the experiments under anaerobic conditions. HL wrote the manuscript with input from all authors.
